# Emotional outcomes in tunisian stroke survivors

**DOI:** 10.1192/j.eurpsy.2021.649

**Published:** 2021-08-13

**Authors:** N. Charfi, S. Elleuch, N. Smaoui, M. Maalej Bouali, L. Zouari, M. Dammak, C. Mhiri, J. Ben Thabet, M. Maalej

**Affiliations:** 1 Psychiatry C Department, Hedi chaker University hospital, sfax, Tunisia; 2 Neurology Departement, Habib Bourguiba hospital university, sfax, Tunisia; 3 Neurology, Habib Bourguiba Hospital, sfax, Tunisia

**Keywords:** Depression, Anxiety, stroke survivors, HAD

## Abstract

**Introduction:**

Depression and anxiety are recognized as common psychiatric complications of stroke, yet little is known about their clinical correlates and their impact on functional outcome.

**Objectives:**

To assess the prevalence of anxiety and depression during the first year post-stroke; To determine their relationships with clinical and functional variables.

**Methods:**

We conducted a cross-sectional study, which included 147 patients, followed for stroke that had occurred over the past year. We used the HAD scale in its Arabic version for screening for anxiety and depression and the modified Rankin scale to assess the degree of disability due to stroke.

**Results:**

Anxiety was detected in 55.1% of patients and depression in 67.3% of them. These emotional disturbances were more common during the first six months post-stroke. Depression was more common among male gender (p=0.003). Older age and more than secondary educational attainment correlated with post-stroke anxiety (p respectively 0.013 and 0.002). Post-stroke anxiety and depression were significantly more common in case of infarcts involving the territory of the Sylvian and the anterior cerebral artery (p respectively 0.01 and 0.001). Depression was significantly associated with the presence of motor deficit on the initial neurological examination (p<0.001) and subsequent neurological sequelae (p<0.001). Anxiety and depression were significant predictors of functional disability during the 12 months post-stroke (p=0.007).

**Conclusions:**

Anxiety and depression impair functional ability after stroke. These data may help identify the patients at greatest risk of poor emotional outcomes and thus help in planning appropriate interventions.

